# Use and Effect of Embodied Conversational Agents for Improving Eating Behavior and Decreasing Loneliness Among Community-Dwelling Older Adults: Randomized Controlled Trial

**DOI:** 10.2196/33974

**Published:** 2022-04-11

**Authors:** Lean L Kramer, Lex van Velsen, Jenna L Clark, Bob C Mulder, Emely de Vet

**Affiliations:** 1 Consumption and Healthy Lifestyles Wageningen University & Research Wageningen Netherlands; 2 eHealth cluster Roessingh Research and Development Enschede Netherlands; 3 Biomedical Signals and Systems Group University of Twente Enschede Netherlands; 4 Center for Advanced Hindsight Duke University Durham, NC United States; 5 Strategic Communication Group Wageningen University & Research Wageningen Netherlands

**Keywords:** eHealth, online intervention, embodied conversational agent, lifestyle change, older adult, user experience, eating habits, eating behavior

## Abstract

**Background:**

Embodied conversational agents (ECAs) have been proposed as a promising interaction modality for the delivery of programs focused on promoting lifestyle changes. However, it is not understood what factors influence the health effects of ECAs or their use.

**Objective:**

We aimed to (1) identify whether ECAs could persuade community-dwelling older adults to change their dietary behavior and whether ECA use could decrease loneliness, (2) test the pathways to these effects, and (3) understand factors influencing the use of ECAs.

**Methods:**

A randomized controlled trial was conducted. The intervention group received access to the PACO service for 8 weeks. The waitlist group started PACO use after waiting for 4 weeks. Two primary outcomes (eating behavior and loneliness) were assessed via online questionnaires at intake, upon joining the waitlist, after 4 weeks, and after 8 weeks. The third primary outcome (use) was assessed via data logs. Secondary outcomes were measured at the same time points, via questionnaires or an optional interview.

**Results:**

In total, 32 participants completed the intervention. We found a significant correlation between use in minutes on the one hand, and perceived usefulness (*r*=0.39, *P*=.03) and enjoyment on the other (*r*=0.38, *P*=.03). However, these did not predict use in the full regression model (*F*_2,29_=1.98, *P*=.16, *R*^2^=0.12). Additionally, PACO use did not lead to improvement in eating behavior (*χ*^2^_2_=0.34, *P*=.85) or a decrease in loneliness (*χ*^2^_2_=0.02, *P*=.99).

**Conclusions:**

Our study did not provide any concluding evidence about factors that are linked to the use or health effects of ECAs. Future service design could benefit from either creating a functional design catering to the predominant stage in the precaution adoption process model of the targeted population, or by personalizing the service based on an intake in which the end user’s stage is determined.

**Trial Registration:**

ClinicalTrials.gov NCT04510883; https://clinicaltrials.gov/ct2/show/NCT04510883

**International Registered Report Identifier (IRRID):**

RR2-10.2196/22186

## Introduction

### Background

Embodied conversational agents (ECAs) have been proposed as a promising interaction modality for the delivery of programs focused on promoting lifestyle changes [1], such as physical activity [2-4] and nutrition [5,6], and preconception care [7,8]. ECAs often have a human-like appearance and communicate via prewritten dialogue. They also have the ability to establish and maintain an empathic relationship by using empathic behavior, both verbal, via text or speech, and nonverbal, via facial and gaze expressions and hand and body gestures [9]. These behaviors may make them more engaging than traditional eHealth interventions [10,11]. Results are promising, as ECA interventions have been found to be easier to use [5] and used more frequently [5,11-13] than interventions without an ECA. Nonetheless, ECA use does decline over time, limiting long-term health effects [1,14-17]. Moreover, it is unknown what factors influence use of an ECA. When designing an ECA, designers are advised to select the right role for the ECA, combine the most important personality characteristics, and use informational, nonjudgmental language [18]. In addition, a scoping review identified which use-related factors were assessed when evaluating the effect of ECAs on promoting healthy lifestyles. These factors included usability and user satisfaction, further specified as factors including liking and trusting the ECA and the desire to continue using the ECA. However, evidence for the effect of these factors on ECA use is limited. Furthermore, there is scarce and inconclusive evidence for the health effects of ECAs and the pathways to these effects [1].

**Figure 1:**
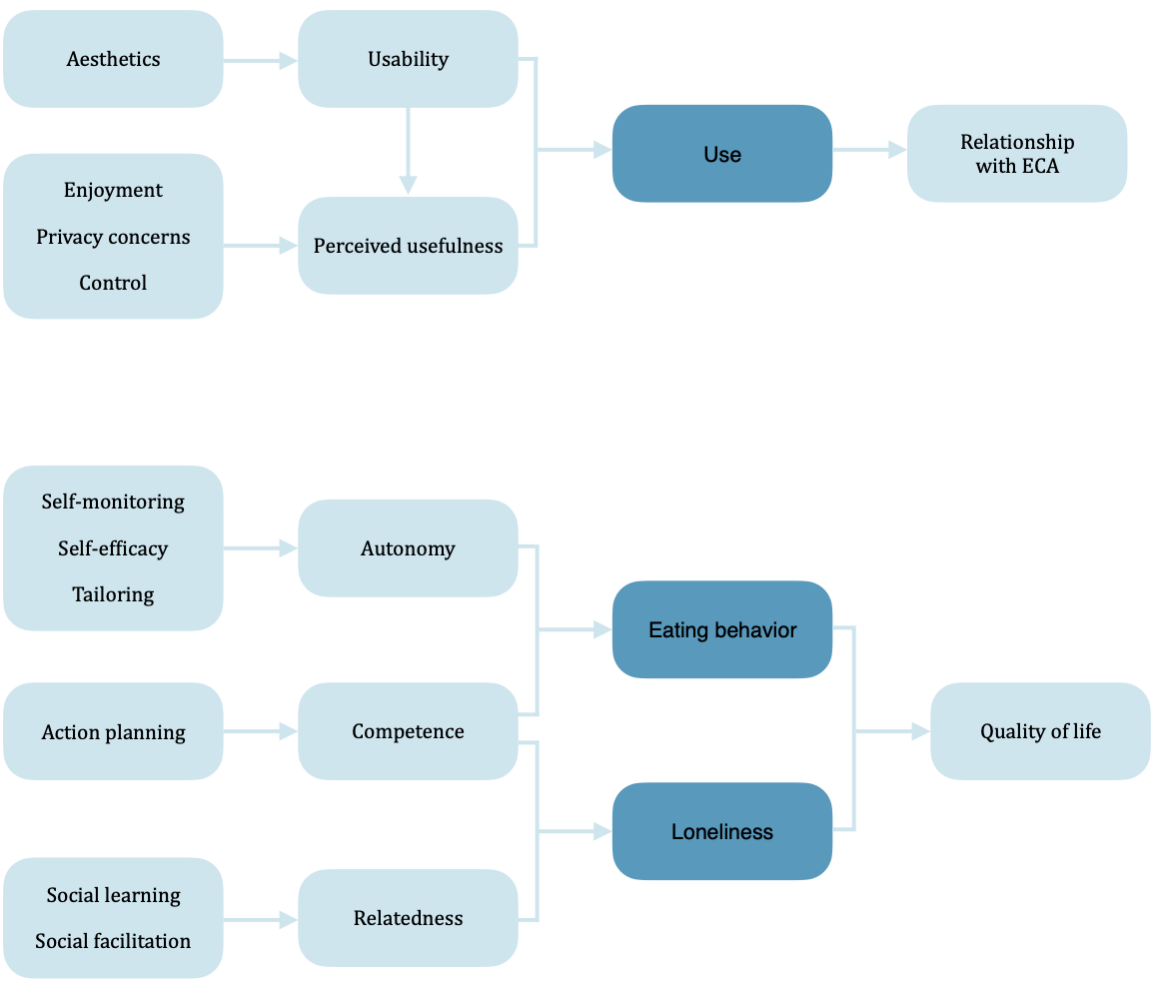
Conceptual models explaining embodied conversational agent (ECA) use and health effects.

In order to assess the pathways to effects and understand ECA use when evaluating an ECA, conceptual models can be used (as shown in Figure 1; further details were reported in the research protocol for this study [16]). The conceptual model explaining ECA use is based on existing human-computer interaction literature, including the technology acceptance model (TAM) [19]. ﻿The key variables in TAM are perceived usefulness and perceived ease of use. Systematic reviews have shown that these 2 variables typically explain about 40 percent of an individual’s intention to use a technology in a variety of contexts [20-22]. However, there is mixed evidence regarding whether intention predicts actual use [23,24]. Since actual use, rather than intention to use, is deemed necessary to achieve any health benefits, use is at the center of the conceptual model. Increased use is expected to improve the intensity of the relationship with the ECA, because of the capacity of ECAs to establish and maintain an empathic relationship. Usability and perceived usefulness are hypothesized to act as antecedents for use, whereas increased usability is expected to result in increased perceived usefulness.

The conceptual model explaining health effects occurring after the use of an ECA starts with behavioral change techniques (see Multimedia Appendix 1), which are expected to lead to an improvement in the 3 basic psychological needs: autonomy, competence, and relatedness. [25]. Ultimately, improved health behaviors will lead to a better quality of life. This model is primarily based on self-determination theory [25] and has an explorative character. By contrast, the classification system of Teixeira et al [26,27] is used to form hypotheses to explain which techniques improve which needs. Hence, the objectives of this study were to (1) identify whether ECAs could persuade community-dwelling older adults to change their dietary behavior and decrease their loneliness, (2) assess the pathways to these effects, and (3) understand ECA use.

## Methods

### Study Design

This study used a randomized controlled trial design. Participants in the first cohort received access to the 8-week intervention immediately, while participants in the second cohort served as a control group, receiving access to the intervention after being placed on a 4-week waiting list.

### Participants and Procedure

We aimed to include a total of 60 participants with a 1:1 ratio of participants per cohort. Participants were deemed eligible if they were aged 65 years or older, not in paid employment, and lived alone and independently at home. In addition, participants needed to speak Dutch, be able to use a tablet or computer by themselves, and have a wireless internet connection. The project members recruited participants via research panels, flyers, newspapers, and social media. After providing informed consent, the participants were invited to complete the intake questionnaire. They were asked to report their demographics (gender, age, educational level, health conditions, risk of malnutrition [28], and eHealth literacy [29]), their possession of a device to use for the study, and their motivation to participate. All participants were asked to complete the baseline questionnaire (T0) after creating an online account and complete another questionnaire after 4 (T1) and 8 (T2) weeks of use. Participants in cohort 2 were asked to complete an additional waitlist questionnaire (Tw) 4 weeks before T0. In the last questionnaire, participants were asked whether they were open to an interview by phone.

### Intervention

**Figure 2:**
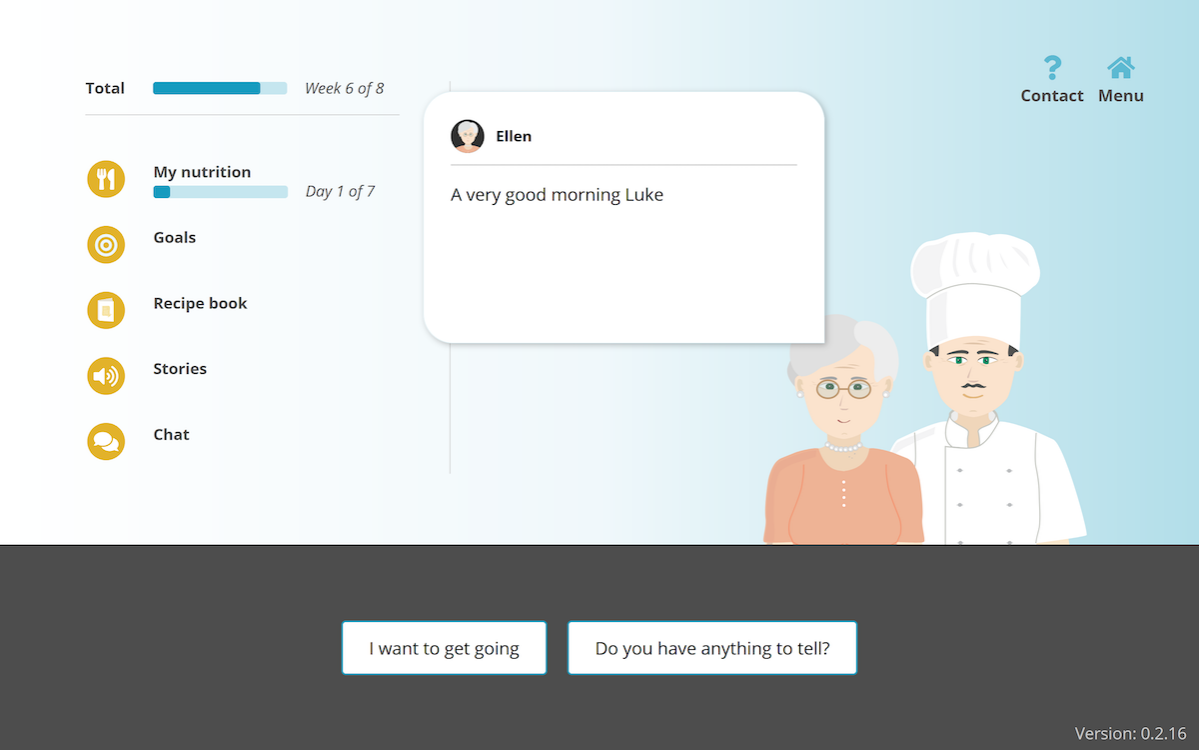
PACO home screen.

The intervention, PACO, is a web-based eHealth service in which 2 ECAs engage in dialogue with an older adult to provide motivation for improving eating behavior and decreasing loneliness. The service consists of 5 modules, each one applying a different behavioral change technique (Figure 2, Multimedia Appendix 1). The user can engage in dialogue with Herman (the cook, who provides nutritional advice) and Ellen (the peer, who provides social advice). The ECAs are represented as 2D humans in cartoon style, are not animated, and use text as the means of communication. During the onboarding process, the ECAs introduce themselves and explain the PACO program.

### Outcomes

The primary outcomes include use of the service, eating behavior, and loneliness. Use was assessed via log data collected on the PACO back end. Eating behavior was self-assessed by 3 open questions about the previous day’s fruit, vegetable, and liquid intake and loneliness was assessed via a validated questionnaire (Table 1 shows further details). The experience and the willingness to pay for PACO were measured via a self-compiled scale. All other outcomes were measured via validated online questionnaires. In an interview of approximately 30 minutes, participants were asked further questions about their experiences with PACO and any behavioral changes.

**Table 1:** Study outcomes measured via questionnaires in each study phase.

Outcome		Scale	Tw	T0	T1	T2
**Use-related outcomes**						
	Relationship with ECA^a^	Rapport scale [30-32]	N/A^b^	✓	✓	✓
	Usability	System usability scale [33]	N/A	N/A	N/A	✓
	Enjoyment	Affect scale [34]	N/A	N/A	N/A	✓
	Aesthetics	Classic aesthetics [35]	N/A	N/A	N/A	✓
	Privacy concerns	Concern for privacy scale [36]	N/A	N/A	N/A	✓
	Control	Active control [37]	N/A	N/A	N/A	✓
	Perceived usefulness	Perceived usefulness scale [19,38]	N/A	N/A	N/A	✓
**Health-related outcomes**						
	Eating behavior	N/A	✓	✓	✓	✓
	Loneliness	De Jong Gierveld loneliness scale [39]	✓	✓	✓	✓
	Quality of life	Brief older people’s quality of life questionnaire [40]	✓	✓	✓	✓
	Autonomy, competence, and relatedness	Basic psychological need satisfaction and frustration scales [41-43]	✓	✓	✓	✓
**Other outcomes**						
	Experience	N/A	N/A	N/A	✓	N/A
	Willingness to pay	N/A	N/A	N/A	N/A	✓

^a^ECA: embodied conversational agent.

^b^N/A: not applicable.

### Data Analyses

We created a single score for each scale and checked the test assumptions. Due to the violation of the linearity assumption, we deviated from the original protocol by using nonparametric tests. Relationships between demographics and the main study outcomes were calculated using Spearman ρ and, for gender, Mann-Whitney *U*. Differences between Tw and T0, and in health-related outcomes over time, were compared using the Friedman test. Differences in the strength of the relationship with the ECA over time were compared with a repeated-measures ANOVA. Spearman ρ was used to calculate the correlations between use- and health-related outcomes. Linear regression analysis was used to calculate the multivariate relationships between use, eating behavior, loneliness, and significant outcomes. The statistical significance level was *P*<.05. Recordings of the interviews were transcribed and thematically analyzed by LLK and BCM.

### Ethics Approval

This study was preregistered at ClinicalTrials.gov (NCT04510883) and approved by the medical ethics committee of Wageningen University (number NL73121.081.20). We refer to the study protocol article for all details on the protocol, the development process of the intervention, and the conceptual models [16].

## Results

### Drop-out, Baseline Characteristics, and Motivation

In total, 51 participants met the inclusion criteria. Nineteen participants did not use the PACO service for 14 consecutive days and were treated as dropouts. Among participants who dropped out, 7 did not respond to emails or telephone calls, 3 dropped out due to illness, 3 due to lack of time, 2 due to lack of motivation, 2 due to difficulties with the service, and 1 due to internet issues. Eight participants who dropped out had created an account, of whom 4 had completed T0. The mean age of the 32 participants was 73.00 years (SD 5.33, range 65-85); 18 (56%) were women. In total, 12 (38%) had completed high school or an associate degree and 19 (59%) had completed college or university. The mean eHealth literacy score was 29.25 (SD 4.36, range 15-34), and the risk of malnutrition was 9.69 (SD 1.35, range 7-11). None of the demographic characteristics were significantly associated with use, eating behavior, or loneliness, and there were no significant differences in health-related outcomes between Tw and T0. During intake, participants stated that they were mainly motivated to participate because they were interested in research and in new developments and thought it was important to contribute. Some participated because they were interested in nutrition and wanted to stay healthy or improve their habits.

### Health Effects

The ECAs were not able to persuade users to change their fruit, vegetable, or liquid intake (*χ*^2^_2_=0.34, *P*=.85) or decrease loneliness (*χ*^2^_2_=0.02, *P*=.99). There were also no significant differences over time in quality of life (*χ*^2^_2_=2.99, *P*=.22), autonomy (*χ*^2^_2_=0.34, *P*=.85), competence (*χ*^2^_2_=2.32, *P*=.31), or relatedness (*χ*^2^_2_=2.46, *P*=.29). Table 2 shows all descriptive health outcomes.

During the interviews, most participants indicated that they thought they had a healthy diet. Nonetheless, a majority mentioned that the food diary helped them to become aware of their food intake. Some people were even shocked by the observation that they had such a fixed eating pattern and described PACO as a wake-up call. About half of the participants mentioned that they did introduce changes into their diet, such as cooking with more fresh ingredients, baking bread, eating more fruits and vegetables, and eating less meat. With respect to loneliness, most participants mentioned that they already had ample social contacts, even though some stated that they were feeling rather lonely. Apart from the unfortunate timing of the pandemic, 4 participants mentioned making changes in their social network because of PACO. For example, 1 participant created a list of everyone he knew and contacted them occasionally. Also, the chat connected a few people with each other and resulted in one-on-one contacts.

**Table 2:** Descriptive health outcomes.

	Scale	Tw, mean (SD)	T0, mean (SD)	T1, mean (SD)	T2, mean (SD)
Eating behavior	0-300	237.04 (45.33)	215.84 (72.12)	215.70 (65.92)	223.01 (71.28)
Loneliness	1-5	2.27 (1.71)	2.47 (1.78)	2.62 (1.91)	2.44 (1.92)
Quality of life	13-65	54.93 (4.93)	56.09 (5.60)	55.47 (6.56)	54.78 (5.85)
Autonomy	1-5	4.11 (0.42)	3.99 (0.54)	4.05 (0.60)	4.07 (0.56)
Competence	1-5	4.24 (0.37)	4.05 (0.58)	4.19 (0.52)	4.22 (0.57)
Relatedness	1-5	4.21 (0.38)	4.33 (0.56)	4.31 (0.53)	4.34 (0.51)

### Pathways to Effects

Following our conceptual model for health, we expected to find a significant correlation between minutes spent on the different modules and eating behavior. However, this was not the case (*P*>.05, see Table 3 for all correlations). With respect to the other pathways, we found that competence correlated with eating behavior (*r*=–0.38, *P*=.03) and that it predicted eating behavior over time (*F*_1,30_=4.30, *P*=.047, *R^2^*=0.13). Quality of life (*r*=–0.60, *P*<.001), autonomy (*r*=–0.38, *P*=.03), relatedness (*r*=–0.59, *P*<.01), and number of chat messages (*r*=0.72, *P*=.03) correlated with loneliness, but did not predict loneliness (*F*_4,8_=1.32, *P*=.40, *R^2^*=0.14).

**Table 3:** Spearman correlations for health-related outcomes and PACO modules.

Variable		Eating behavior	Loneliness	Quality of life	Autonomy	Competence	Relatedness	Food diary	Goals	Recipes	Stories	Chat
**Eating behavior**												
	Spearman correlation	1	0.10	–0.21	–0.11	–0.38	–0.01	0.19	0.12	–0.27	0.04	0.01
	*P* value	—^a^	.57	.28	.57	.03	.94	.30	.53	.13	.81	1.00
**Loneliness**												
	Spearman correlation	0.10	1	–0.60	–0.38	–0.16	–0.59	0.01	–0.09	–0.23	–0.13	0.72
	*P* value	.57	—	<.001	.03	.39	<.001	.98	.62	.21	.48	.03
**Quality of life**												
	Spearman correlation	–0.21	–0.60	1	0.75	0.47	0.67	0.13	0.20	0.14	0.36	–0.45
	*P* value	.26	<.001	—	<.001	.007	<.001	.48	.28	.45	.046	.22
**Autonomy**												
	Spearman correlation	–0.11	–0.38	0.75	1	0.55	0.60	0.09	–0.02	–0.03	0.16	–0.31
	*P* value	.57	.03	<.001	—	.001	<.001	.62	.92	.88	.37	.41
**Competence**												
	Spearman correlation	–0.38	–0.16	0.47	0.55	1	0.43	0.06	–0.32	–0.24	–0.01	0.06
	*P* value	.03	.39	.007	.001	—	.014	.74	.08	.18	.99	.89
**Relatedness**												
	Spearman correlation	–0.01	–0.59	0.67	0.60	0.43	1	0.12	0.13	0.20	0.24	–0.29
	*P* value	.94	<.001	<.001	<.001	.01	—	.51	.49	.28	.20	.45
**Food diary**												
	Spearman correlation	0.19	0.01	0.13	0.09	0.06	0.12	1	0.02	–0.14	–0.13	–0.27
	*P* value	.30	.98	.48	.62	.74	.51	—	.92	.46	.49	.48
**Goals**												
	Spearman correlation	0.12	–0.09	0.20	–0.02	–0.32	0.13	0.02	1	0.34	0.44	–0.37
	*P* value	.53	.62	.28	.92	.08	.49	.92	—	.06	.01	.33
**Recipes**												
	Spearman correlation	–0.27	–0.23	0.14	–0.03	–0.24	0.20	–0.14	0.34	1	0.11	–0.21
	*P* value	.13	.21	.45	.88	.18	.28	.46	.06	—	.54	.59
**Stories**												
	Spearman correlation	0.04	–0.13	0.36	0.16	–0.01	0.24	–0.13	0.44	0.11	1	0.03
	*P* value	.81	.48	.046	.37	.99	.20	.49	.01	.54	—	.93
**Chat**												
	Spearman correlation	0.01	0.72	–0.45	–0.31	0.06	–0.29	–0.27	–0.37	–0.21	0.03	1
	*P* value	1.00	.03	.22	.41	.89	.45	.48	.33	.59	.93	—

^a^Not applicable.

### Understanding ECA Use

#### Use of PACO and Trends Over Time

**Figure 3:**
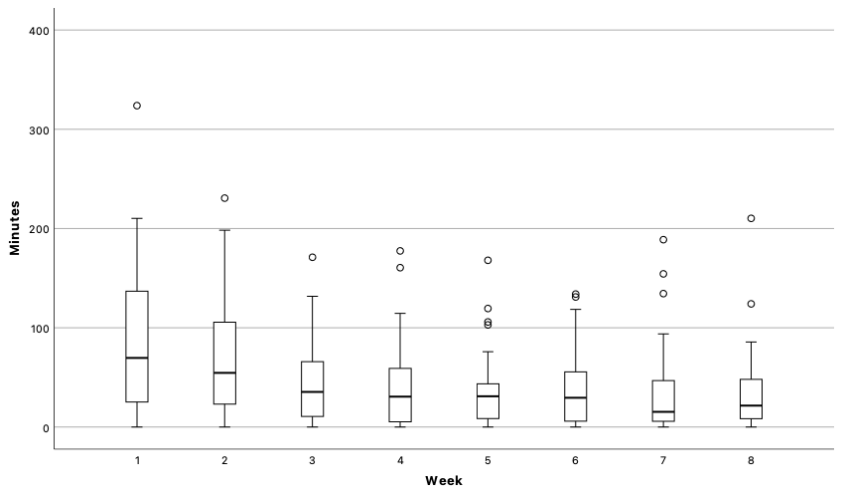
Minutes per week.

On average, participants logged in 39.97 times (SD 37.38, range 10-197). Minutes per week decreased from a median of 69.66 in week 1 to 21.57 minutes in week 8 (Figure 3). The Friedman test confirmed this decline over time, showing a significant difference in use between weeks (*χ*^2^_7_=31.46, *P*<.001). The median time for using PACO was 15 h, 15 min, and 05 s. The average total time spent on PACO was 6 h, 30 min (SD 05 h, 54 min, 01 s), and the time spent per session was 11 h, 10 min (SD 05 h, 44 min). The average number of modules used per session was 2.39 (SD 0.34). The most time was spent on the food diary (85.45%), followed by the recipes (6.36%), goals (4.58%), and stories (3.61%). In total, 11 participants signed up for the chat. They sent a mean of 27.78 messages (SD 15.55, range 13-67). During the final interaction, the module used most often was the food diary (41.67%), followed by the chat (25.00%), goals (16.67%), recipes (12.50%), and stories (4.17%).

#### Use-Related Outcomes

Usability, aesthetics, privacy concerns, and perceived control were rated above the midpoint of the scale (Table 4). The enjoyment and usefulness of PACO were rated below the midpoint of the scale, and perceived usefulness was rated relatively low. In total, 30 (94%) participants indicated that they were not willing to pay for PACO. With respect to the amount they would be willing to pay, results were contradictory, with 28 (88%) not willing to pay anything, and 4 (13%) willing to pay €5 (US $5.50). Following our conceptual model for ECA use, we found that aesthetics correlated significantly with usability (*r*=0.44, *P*=.01) and enjoyment correlated with perceived usefulness (*r*=0.48, *P*=.005). Although we found that perceived usefulness (*r*=0.39, *P*=.03) and enjoyment (*r*=0.38, *P*=.03) correlated with use in minutes (Table 5 shows all correlations), in the full regression model, these did not predict use (*F*_2,29_=1.98, *P*=.16, *R^2^*=0.12).

During the interviews, participants stated that although they read the module content, they did not truly engage and often reported that the content was not helpful. For example, participants listened to stories and read recipes, but did not act. In some cases, this was due to issues of tone, such as storytellers being seen as patronizing or the discomfort of endorsing dining alone. In other cases, such as the chat, participants simply did not wish to speak to people they did not know, or, if they did do so, the conversations felt shallow.

**Table 4:** Descriptive use outcomes.

	Scale	Outcome, mean (SD)
Usability	0-100	64.53 (17.98)
Enjoyment	1-7	3.26 (0.81)
Aesthetics	1-7	4.82 (1.21)
Privacy concerns	1-7	5.14 (1.28)
Control	1-7	4.78 (1.20)
Perceived usefulness	1-7	2.56 (0.99)

**Table 5:** Spearman correlations for use and use-related outcomes.

Variable		Use	Relationship with ECA^a^	Usability	Perceived usefulness	Aesthetics	Enjoyment	Privacy concerns	Control
**Use**									
	Spearman correlation	1	–0.13	–0.05	0.39	0.34	0.38	0.30	–0.01
	*P* value	—^b^	.47	.80	.03	.06	.03	.09	.99
**Relationship with ECA**									
	Spearman correlation	–0.13	1	–0.01	0.31	0.28	0.28	0.01	0.32
	*P* value	.47	—	.96	.08	.13	.12	.96	.07
**Usability**									
	Spearman correlation	–0.05	–.01	1	–0.13	0.44	0.23	0.35	0.48
	*P* value	.80	.96	—	.47	.01	.21	.05	.005
**Perceived usefulness**									
	Spearman correlation	0.39	0.31	–0.13	1	0.27	0.48	0.09	0.01
	*P* value	.03	.08	.47	—	.13	.005	.64	.95
**Aesthetics**									
	Spearman correlation	0.34	0.28	0.44	0.27	1	0.78	0.54	0.51
	*P* value	.06	.13	.01	.13	—	<.001	.001	.003
**Enjoyment**									
	Spearman correlation	0.38	0.28	0.23	0.48	0.78	1	0.32	0.38
	*P* value	.03	.12	.21	.005	<.001	—	.07	.04
**Privacy concerns**									
	Spearman correlation	0.30	0.01	0.35	0.09	0.54	0.32	1	0.48
	*P* value	.09	.96	.05	.64	.001	.07	—	.005
**Control**									
	Spearman correlation	–0.01	0.32	0.48	0.01	0.51	0.38	0.48	1
	*P* value	.99	.07	.005	.95	.003	.04	.005	—

^a^ECA: embodied conversational agent.

^b^Not applicable.

#### Relationship With the ECAs

**Figure 4:**
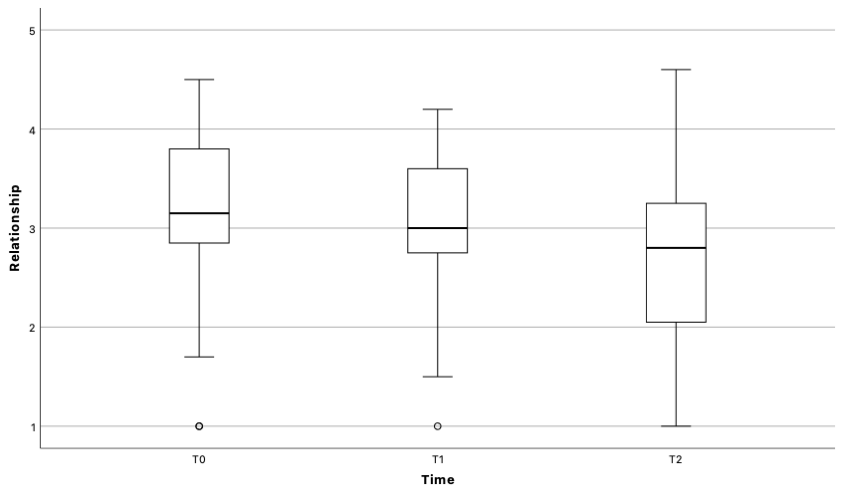
Relationship with the embodied conversational agents over time.

The strength of the relationship with the ECAs decreased over time (*F*_1.72,53.33_=4.22, *P*=.02; more details shown in Figure 4). Posthoc analysis with Bonferroni correction showed that the difference between T1 and T2 was significant (*P*=.047). Contrary to our expectation, the relationship did not correlate with use (*r*=–0.13, *P*=.47).

During the interviews, most participants were neutral about the ECAs, or reported not having noticed them. Six participants mentioned that the ECAs made PACO easier to use, more engaging, or more enjoyable compared to plain text, or even described them as “fantastic.” On the other hand, 3 participants found the ECAs to be childish and unreal and the participants considered themselves too rational to regard the ECAs as actual people.

## Discussion

This study used a randomized controlled trial to investigate the effectiveness, the pathways to effects, and the mechanisms that underlay the use of an ECA targeting eating behavior and loneliness among older adults. The results showed that neither the ease of use of the PACO service nor the user experience explained the extent to which it was used. Furthermore, the use of PACO did not result in improved fruit, vegetable, or liquid intake or reduced loneliness. Our findings might, on first sight, contradict our hypotheses, and add to the mixed evidence base on nutritional ECAs [5,6,44]. On the other hand, we can also take these results as valuable lessons for the future design of eHealth services.

Participants did become more aware of their eating behavior due to the self-monitoring tool, and thus also became more aware of behaviors they could improve. In the terms used in the precaution adoption process model (PAPM) [45], they were “deciding about acting.” However, in the interviews, participants expressed high self-perceived health and no need for change. This suggests users might well have been in the stage of “decided not to act.” It is known that the PAPM stage plays a significant role in the perceived persuasiveness of different behavioral change techniques [46]. Hence, different needs should have been nurtured in our participants, as they were still in an earlier stage of the model. If this was the case, then the design of future services could benefit from either creating a functional design catering toward the predominant stage of the targeted population or personalizing the service based on an intake process that considers the stage of the end user.

To our knowledge, we are among the first to study factors that help understand ECA use. Surprisingly, we found that the use of PACO could not be explained by its usability, privacy concerns, perceived usefulness, or level of enjoyment. Furthermore, positive ratings on aesthetics and perceived control were not associated with time spent using PACO, although these factors did have a positive correlation with usability. Instead of arguing that these factors are not relevant for the development of an ECA, we argue that a certain threshold might be necessary for a service to be used. This is in line with other work on ECAs among older adults, which has shown that technical problems have a negative impact on use, adaptiveness, usefulness, and trust [47]. It has yet to be determined what factors positively influence the use of an ECA. Instead of focusing on traditional use-related factors, as we did in our conceptual model explaining ECA use, future research might benefit from examining certain threshold scores in earlier stages of development. Furthermore, the PAPM might help understand ECA use. If users do not intend to change their behavior, for example, it can be expected that they will not engage with the ECA. Research has indeed shown that this is true for the adoption of nutrition and fitness apps among the general population [48].

This study has limitations. First, we received a very low response to our flyers, social media posts, and advertorials. Newspaper interviews and phone calls to potential participants by the research panels resulted in a greater, yet still limited, response. Because of this nonresponse, we do not know why more older adults did not want to participate in this study. In total, 5 potential participants indicated that they did not want to participate on the consent form. One unintended effect of this method of obtaining informed consent could have been that people who were unable to provide consent were excluded. As a result of the small sample size, the overall power of this study was low. We consider that not measuring the PAPM stage was a second limitation of this study. We suspect that PAPM stage is a factor that might provide more insight into both the use and effectiveness of the service (ie, participants who use the service more frequently and report health-related effects might be more likely to act). Finally, the ongoing COVID-19 pandemic might have influenced our results. We did rewrite the content of PACO to match the current situation and focused on online alternatives for engaging in social interactions. Nonetheless, participants felt they were not able to be more socially active due to government restrictions. Indeed, loneliness increased in our target group during the pandemic [49]. This might have counteracted the decrease in feelings of loneliness that we expected.

In conclusion, this study illustrates how to use a conceptual model to guide the evaluation of an ECA service in terms of both its level of use and its health effects, although it did not provide us with any conclusive evidence of its actual effectiveness. Nonetheless, our results provide valuable directions for future studies in this emerging field.

## Appendix

Multimedia Appendix 1 The modules of the PACO service.

Multimedia Appendix 2 CONSORT e-HEALTH checklist (V 1.6.1).

## Abbreviations

ECA: embodied conversational agent
PAPM: precaution adoption process model
TAM: technology acceptance model
