# Supplementary material for: Use and Effect of Embodied Conversational Agents for Improving Eating Behavior and Decreasing Loneliness Among Community-Dwelling Older Adults: Randomized Controlled Trial
Source: JMIR Form Res. 2022 Apr 11;6(4):e33974. doi: 10.2196/33974 (PMC9039822; doi:10.2196/33974)
Supplement: Multimedia Appendix 1 [file formative_v6i4e33974_app1.docx]

## Multimedia Appendix 1

**Table 1.** The modules of the PACO service.

| Week | Module | Behavior change technique | SDT component, target behavior | Rationale |
| --- | --- | --- | --- | --- |
| 1 | Food diary | Self-monitoring | Autonomy, eating behavior | Users record what they have eaten, with whom, and how they appreciated the meal. There is an option to set reminders. When users know what they eat and drink, we aim to give them the feeling that they are able to change their behavior[47], leading to an actual change in eating behavior. |
| 2-8 | Goals | Action planning | Competence, eating behavior and loneliness | Users can choose from a list of social and eating goals. Via dialogue, Ellen explains the goal and provides tips. Users create a personal action plan and track their progress, with the option to set reminders. When users carry out their plans, we aim to improve their feelings of competence[25], leading to change in eating behavior and feelings of loneliness. |
| 1-8 | Recipes | Tailoring and self-efficacy | Autonomy, eating behavior | Via dialogue, Herman helps users to select a healthy and easy-to-prepare recipe (>280), based on users’ dietary wishes and preferences. By helping users cook their own meals, in line with their preferences, we hope to increase feelings of autonomy via self-efficacy[48], leading to a change in eating behavior. |
| 1-8 | Stories | Social learning | Relatedness, loneliness | Users can listen to stories from other older adults about physical and/or virtual social activities they perform. Ellen can provide more information on the activity. When users learn from each other, we hope that they will feel more related to others and have fewer feelings of loneliness[49], [50]. |
| 1-8 | Chat | Social facilitation (peer support) | Relatedness, loneliness | Via WhatsApp, users can interact with one another. Ellen is also included and asks questions. When users interact with one another, we hope that they will experience increased feelings of relatedness and decreased feelings of loneliness[51], [52]. |
